# Differences and Similarities in Neuropathy in Type 1 and 2 Diabetes: A Systematic Review

**DOI:** 10.3390/jpm11030230

**Published:** 2021-03-22

**Authors:** Mar Sempere-Bigorra, Iván Julián-Rochina, Omar Cauli

**Affiliations:** 1 Department of Nursing, Faculty of Nursing and Podiatry, University of Valencia, 46010 Valencia, Spain; marsembi@outlook.es (M.S.-B.); ivan.julian@uv.es (I.J.-R.); 2 Frailty Research Organized Group (FROG), Department of Nursing, University of Valencia, 46010 Valencia, Spain

**Keywords:** cytokines, vibratory sensitivity, thermal sensitivity, muscle, biomarkers, insulin

## Abstract

Background: Diabetic neuropathy is defined as the dysfunction of the peripheral nervous system in diabetic patients. It is considered a microvascular complication of diabetes mellitus. Its presence is associated with increased morbidity and mortality. Although several studies have found alterations at somatic motor, sensory levels and at the level of autonomic nervous system in diabetic patients, there is not a systematic approach regarding the differences in neuropathy between the major variants of diabetes, e.g., type 1 and 2 diabetes at both neurological and molecular level. Data sources: we systematically (Medline, Scopus, and Cochrane databases) evaluated the literature related to the difference of neuropathy in type 1 and 2 diabetes, differences in molecular biomarkers. Study characteristics: seventeen articles were selected based on pre-defined eligibility criteria. Conclusions: both superficial sensitivity (primarily thermal sensitivity to cold) and deep sensitivity (such as vibratory sensitivity), have been reported mainly in type 2 diabetes. Cardiac autonomic neuropathy is one of the diabetic complications with the greatest impact at a clinical level but is nevertheless one of the most underdiagnosed. While for type 1 diabetes patients most neuropathy alterations have been reported for the Valsalva maneuver and for the lying-to-standing test, for type 2 diabetes patients, alterations have been reported for deep-breathing test and the Valsalva test. In addition, there is a greater sympathetic than parasympathetic impairment, as indicated by the screening tests for autonomic cardiac neuropathy. Regarding subclinical inflammation markers, patients with type 2 diabetes showed higher blood levels of inflammatory markers such as high-sensitivity C-reactive protein, proinflammatory cytokines IL-6, IL-18, soluble cell adhesion molecules and E-selectin and ICAM-1, than in type 1 diabetes patients. By contrast, the blood levels of adiponectin, an adipocyte-derived protein with multiple paracrine and endocrine activities (anti-inflammatory, insulin-sensitizing and proangiogenic effects) are higher in type 1 than in type 2 diabetic patients. This review provides new insights into the clinical differences in type 1 and 2 diabetes and provide future directions in this research field.

## 1. Introduction

Diabetes is a chronic, metabolic disease characterized by elevated levels of blood glucose, which leads over time to serious damage to the heart, blood vessels, eyes, kidneys, and nerves. Its consequences are one of the most serious public health problems with a considerable impact on both human and health resources. Diabetes not only affects the individual’s functionality and quality of life, it is also associated with premature mortality as well as increased morbidity [1]. There are several forms of diabetes [2,3,4]. Type 1 diabetes is a chronic autoimmune disease characterized by the pancreas losing the ability to generate insulin, the hormone that regulates hyperglycemia. For this reason, people with type 1 diabetes will require daily insulin administration for life. Type 2 diabetes is characterized by the presence of high blood glucose levels due to the body’s resistance to insulin, which means that although this hormone is present in the circulation, the cells cannot use it properly to introduce the sugar into their interior. The main causes of insulin resistance are lack of physical activity and the accumulation of adipose tissue, so excess weight and a sedentary lifestyle seem to be the main risk factors for the development of this disease. Maturity Onset Diabetes in the Young (MODY) is caused by genetic defects of the pancreatic beta cells due to different genetic alterations, presented as autosomal dominant inherited disorders leading to a defect in insulin secretion. Cystic fibrosis related diabetes is a genetic disease that affects multiple organs including the pancreas, which can lead to the development of diabetes. Diabetes can be secondary to the effects of some medications (for examples glucocorticoids and immunosuppressants drugs) that can alter the secretion or action of insulin. Pregnancy puts a great metabolic strain on the mother’s body, which can sometimes lead to some insulin resistance. As a result, the pancreas has to produce more insulin to get glucose into the cells and reduce its accumulation in the blood, and sometimes this organ is unable to secrete enough of the hormone, so blood glucose levels rise leading to gestational diabetes and glucose intolerance resembling type 2 diabetes. The most prevalent types of diabetes are type 1 and 2, but type 2 diabetes is up to 10 times more frequent than type 1 diabetes. The incidence of type 1 diabetes increases with age until it reaches its peak at around 10 to 14 years of age, but the disease can debut at any age [5]. Meanwhile, the peak of the incidence of type 2 diabetes is later, at between 55 and 59 years old [6]. Type 1 and 2 diabetes show a slightly higher prevalence in men than in women. An estimated 462 million people worldwide, the equivalent to 6.28% of the world’s population, are affected by type 2 diabetes. The global incidence of type 1 diabetes began to rise in the 1950s with an average annual increase of 3–4% over the past three decades, and incidences of approximately 10–20 cases per 100,000 population in southern Europe and the United States of America [1,6]. Healthcare expenditure on diabetes care is at least 3.2 times higher than the calculated average per capita and increases to 9.4 times in the presence of complications. In terms of functionality, diabetes is among the top 10 disabling diseases [5], and among its diabetic complications, neuropathy affects quality of life and is associated with an increased risk of amputations [7,8], morbidity and mortality [9,10]. Diabetic peripheral neuropathy is the most common form of neuropathy worldwide. The most recent studies in Europe and the United States of America indicate a prevalence of diabetic peripheral neuropathy ranging from 6 to 51% of the population of people with diabetes. The prevalence of diabetic peripheral neuropathy in adults with type 1 diabetes is 6% at the onset of the disease, increasing to 30% after 13–14 years of progression [11,12]. This prevalence is somewhat higher among people with type 2 diabetes and is present in 26% of young people with type 2 diabetes. Among adults with diabetes, about 40% of patients at cardiovascular risk have peripheral neuropathy [11].

Risk factors for the development of neuropathy include different variables such as age, duration of diabetes, glycemic control, and comorbidities such as hypercholesterolemia, hypertension, obesity, and smoking. With regard to diabetic neuropathy, studies reporting the prevalence of peripheral neuropathy in young people with diabetes have determined rates of 7% in patients with type 1 diabetes and 22% with type 2 diabetes [13].

These data suggest a difference in the pathophysiological mechanisms of diabetic neuropathy between patients with type 1 and type 2 diabetes. Given the characterization of the eventual differences between neuropathy in people with type 1 and type 2 diabetes, it is important to know the best tool to assess them, in order to treat and prevent their adverse effects, and to be able to plan specific care depending on the manifestations of neuropathy that may occur in the two most frequent types of diabetes. This review aims to clarify these aspects, which we believe to be fundamental in an individualized approach to diabetes neuropathy, biomarkers investigations and appropriated interventions at a clinical and community level, depending on the type of patient with diabetes being treated.

## 2. Materials and Methods

This study was designed and developed according to the Preferred Reporting Items for Systematic Reviews and Meta-Analyses (PRISMA) [14] guidelines, which represent an evidence-based minimum set of items aimed at assessing the benefits and harms of a healthcare intervention.

### 2.1. Literature Search

We searched the literature in multiple electronic bibliographic databases (Medline, Scopus, and Cochrane), covering all entries until 31 July 2020. The reference lists of all the relevant articles were manually cross-referenced in order to identify additional articles. The primary search terms used were “neuropathy” and “diabetes”, with one of the following terms: “type I and II (or 1 and 2)”, “difference”, “gender/sex differences”, “sensory”, “motor”, “autonomic”. We used the Medical Subject Headings (MeSH) thesaurus to find standard descriptors and their synonyms related to the area of knowledge to be studied. We used these terms to construct the search equation, which was made up of three main components that were semantically related to many other descriptors that were included in the equation. These three parts of the equation were: autonomy * neuropathy, somatic neuropathy, and diabetes mellitus, and they were joined by the Boolean operator “AND”, with their related terms connected by the operator “OR”.

### 2.2. Inclusion and Exclusion Criteria

We applied the following inclusion criteria in order to answer the research question: (1) acknowledged as an original article including reports with different experimental designs such as experimental studies (randomized and placebo-controlled trials) and observational studies (cohort and cross-sectional studies, case series, and case–control studies); (2) full text published in either English or Spanish; (3) neuropathy was specifically assessed in the Methods section by physiological assessments and/or specific neuropathy questionnaires; (4) the authors separately analyzed the outcomes in type 1 and type 2 diabetic patients. 

### 2.3. Data Collection and Analysis

The database search results were uploaded into a web-based system which was used to manage the screening process, and duplicate citations were removed. To determine which studies would be included, three members of the review team independently screened the title and abstracts of the articles extracted from the literature search. The electronic full text was retrieved for studies on which the reviewers agreed, based on our inclusion/exclusion criteria. Two reviewers independently extracted the following data for each of these articles: the country where the study was conducted, the number of participants, the age of participants at the time of inclusion, their sex, the assessment tools for evaluating neuropathy, the differences between type 1 and 2 diabetic patients, and the main outcomes: sensory, motor, and autonomic neuropathy, biomarkers and effects of age and gender. Any disagreement between the two reviewers regarding the papers and data extracted from them was resolved by the third author.

## 3. Results

**Figure 1:**
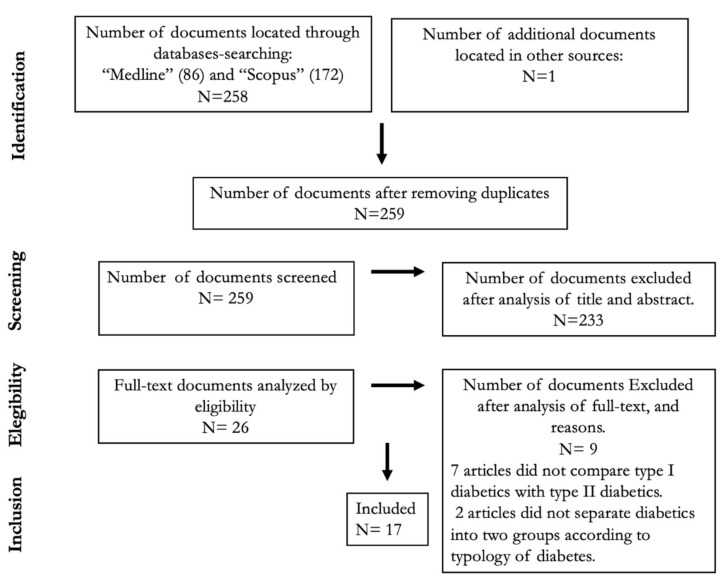
Systematic review workflow.

A total of 259 papers were retrieved from the studies identified by our search strategy; after eliminating duplicates, 26 met the inclusion criteria and were analyzed in detail; finally, 17 articles fulfilled the criteria and aims of this review (Figure 1). Most articles were excluded because they did not compare the presence and/or severity of neuropathy between type 1 and 2 diabetes. Two researchers independently summarized the results which emerged from this literature review under five headings: (1) Differences in sensory neuropathy; (2) Differences in motor neuropathy; (3) Differences in autonomic neuropathy; (4) Differences in markers associated with neuropathy in type 1 and 2 diabetes; (5) Effect of age and sex on neuropathy in type 1 and 2 diabetes.

The most important variables of the selected studies are summarized in Table 1, Table 2 and Table 3, and their results are shown and discussed in the respective sections.

### 3.1. Sensory Neuropathy

The main characteristics of the studies comparing type 1 and 2 diabetic patients regarding impairment of sensitivity are shown in Table 1. Regarding the thermal-analgesic sensitivity which shares common anatomical routes (superficial sensitivity) three studies found thermal sensitivity is more impaired in type 2 patients than in type 1 patients [17,19,21]. Cold sensitivity impairment was higher in type 2 patients than in type 1 patients (60% versus 40%, respectively), whereas impairment in warm sensitivity was only found in 10% of type 1 patients and in no type 2 diabetic patients [17]. No significant differences were found between type 1 and 2 patients for cold and heat sensitivity [33]. Impairment of pain sensitivity was not reported in any group of type 1 and 2 diabetic patients in one study [17]. For deep sensitivity, impairment in palestesic (vibratory) sensitivity recorded in the malleolus and hallux was higher in type 2 than in type 1 diabetic patients, and was demonstrated across a varied age range (mean age 15–62 years old) [15,17,19,33]. The presence of the palestesic sensitivity, defined as being more than 2 standard deviations below the mean value in normal controls, was found in 40% of type 2 diabetics and in 12% approximatively of type 1 diabetic patients [17], and not found in type 1 diabetics [33] thus reinforcing the data on higher threshold values in type 2 than in type 1 patients. This higher threshold for vibration sensitivity is accompanied by a lower amplitude and conduction velocity of the sural sensory nerve, present in both types of diabetes without any differences [15]. In other studies, the nerve conduction rate in some sensory nerves, e.g., the median nerve (but not in other nerves such as the ulnar and sural nerves) was lower in type 2 than in type 1 diabetes patients [21].

### 3.2. Motor Neuropathy

The main characteristics of studies comparing somatic motor neuropathy at the level of skeletal muscles in type 1 and 2 diabetic patients are shown in Table 2. Balducci et al. [15] evaluated muscle strength by isometric contractions by measuring the maximal voluntary contractions performed at the shoulder press along the sagittal plan, and between the upper arm and the trunk, respectively, for the upper body, and at the leg extension machine at the knee and the hip, for the lower body. No significant association with any type of diabetes was found in the multivariate model when taking into account confounders influencing muscle strength such as age, sex, and physical activity level. Among neuro-electrophysiological alterations, only motor and sensory nerve amplitudes measured in the peroneal motor nerve and sural sensory nerve, respectively, correlate with muscle strength when corrected for the confounding factors mentioned above. In contrast, the conduction velocities and distal latencies are not associated with muscle strength, and all these data suggest that in the early stages, diabetic polyneuropathy mainly consists of axonal damage, whereas later myelin involvement accounts for reduced conduction velocity and latency, which is observed only in specific cases or at later stages. The motor nerve conduction velocity measured in the median and peroneal motor nerves (with the ulnar motor nerve tending towards significant) was significantly lower in type 2 than in type 1 diabetic patients [21].

However, Balducci and co-workers [15] did not detect any difference between type 1 and 2 diabetic patients for velocity and latency measured in the peroneal motor nerves, whereas a tendency (*p* = 0.08) was observed for the amplitude of peroneal motor nerve. The difference in the mean age of participants belonging to the type 1 diabetes group may have played a role in this difference in motor nerve velocity in these two studies (the mean age in Balducci’s study was 46 years, and in Scamarek’s study [21] it was 61 years for the group of patients with type 1 diabetes, whereas the mean age in both studies for diabetic type 2 patients was 66 years). In the upper body motor nerves, Arnold et al. [24] evaluated the compound muscle action potentials from the abductor pollicis brevis muscle using surface electrodes following stimulation of the median nerve at the wrist. All patients (type 1 and 2 diabetes patients, age-, sex-, HbA1c level- and disease-duration-matched) had normal sural nerve conduction amplitudes, and none of them reported neuropathic symptoms or demonstrated any clinical evidence of neuropathy as quantified by pin prick and vibration sensitivity or ankle-deep tendon reflexes. Only the type 1 patients showed abnormalities in nerve excitability recordings, consisting of reductions in the prolongation of the relative refractory period and reductions in superexcitability, and this pattern was probably due to a pattern of axonal depolarization as a result of either a dysfunction of the energy-dependent axonal Na+/K+ pump in isolation or due to microvascular perfusion deficits leading to ischemia. This study provides evidence that altered axonal function precedes the onset of clinical neuropathy in type 1 diabetes.

### 3.3. Autonomic Neuropathy

The main characteristics of studies comparing type 1 and 2 diabetic patients regarding neuropathy at the level of the autonomic (sympathetic and parasympathetic) nervous system in the skeletal muscles are shown in Table 3. Heart rate variability was the most extensively investigated parameter in those studies, comparing type 1 and type 2 cardiac dysautonomy [29,36,37]. Autonomic cardiac neuropathy is diagnosed when there are two or more abnormal results of autonomic function tests [38] and its prevalence is high in diabetic patients, ranging between 20 and 60% [28,29,31,39]. The prevalence of cardiac autonomous neuropathy has been reported either to be higher in type 2 diabetic patients [29,40,41] or similar in both types of diabetes in other reports [31,32,35,42]. Regarding the component of autonomic nervous system, it seems to be affected more by the parasympathetic rather than the sympathetic one [31,39,41].

However, a careful analysis of the tools used to measure cardiac autonomous neuropathy revealed the differences according to the test used to assess it. Heart rate response related to parasympathetic system activation following deep breathing and standing are higher (more impaired) in type 1 than in type 2 patients [15,17], as well as the heart rate variation at rest [17,19]. In contrast, heart rate variability in response to the Valsalva maneuver and the dispersion of QTc interval (QT interval corrected for heart rate) in the electrocardiogram was more impaired in type 2 diabetic patients [17,33]. A combination of the Valsalva and lying-to-standing tests provided the highest sensitivity for detecting cardiac autonomous neuropathy in type 1 diabetic patients (sensitivity = 97.6%, AUC = 0.887) while for type 2 diabetes patients, the deep-breathing and Valsalva test had the highest values (sensitivity = 83.6%, AUC = 0.856) [28]. There were associations between cardiac autonomic neuropathy and long-term complications of diabetes [31,32,35]. Alterations of heart variability during exercise were observed in type 1 and 2 patients with diabetic retinopathy, e.g., less increase in heart rate during exercise and lower recovery rates in patients with diabetic retinopathy, although the pattern of those changes was more impaired in type 1 diabetic patients [35]. There were significant associations between cardiac autonomic neuropathy and hypertension, and between the cardiac autonomic neuropathy/hypertension profile and micro- and macro-angiopathic complications of diabetes [31] and the prolongation of the QTc interval in electrocardiograms [32,33]. Erectile dysfunction (ED) is a common complication of diabetes, and an important cause of decreased quality of life in diabetic men, and its prevalence ranges from 20 to 70% [26,27,43,44,45]. The presence of autonomic neuropathy is one of the pathophysiological hallmarks for erectile dysfunction in diabetes [46,47]. Erectile dysfunction affects both types of diabetes, but it is more prevalent in type 2 patients after controlling for age, but the opposite may be true when other concomitant long-term diabetic complications are present [26,27], and in patients (type 1 and 2) with other diabetic complications (neuropathy, vascular diseases, retinopathy, and nephropathy as well as hypercholesterolemia and hypertension) [26,45]. The existence of a diabetic neuropathy affecting stomach and gallbladder function in diabetic patients has been investigated by means of ultrasound techniques [33,34]. Gastroparesis is a frequent complication of diabetes, and autonomic neuropathy seems to be a cornerstone pathophysiological feature [48]. Gastric emptying is more impaired in type 2 than in type 1 diabetic patients, and in both types, it correlates with cardiac autonomic neuropathy (by means of electrocardiogram QTc segment dispersions) and long-term complications such as diabetic retinopathy and nephropathy [33]. In all diabetic individuals, gallbladder volume progressively decreased after ingestion of the test meal, while the residual volumes were larger in diabetics than in healthy controls. However, there were no significant differences in gallbladder motor function parameters between type 1 and type 2 patients, but compared to healthy subjects, subjects with autonomic neuropathy did exhibit a larger residual gallbladder volume and less complete emptying with earlier gallbladder refilling [34,49].

### 3.4. Differences in Molecular Markers Associated with Neuropathy in Type 1 and 2 Diabetes

The pathophysiology mechanisms underlying neuropathy can develop differently in the two types of diabetes, with different associations between the biomarkers. Regarding subclinical inflammation markers, patients with type 2 diabetes showed higher blood levels of inflammatory markers such as high-sensitivity C-reactive protein, pro-inflammatory cytokines IL-6, IL-18, soluble cell adhesion molecules and E-selectin and ICAM-1, than in type 1 diabetes patients [19,21]. By contrast, the blood levels of adiponectin, an adipocyte-derived protein with multiple paracrine and endocrine activities (anti-inflammatory, insulin-sensitizing and proangiogenic effects) are higher in type 1 than in type 2 diabetic patients [21]. The associations between biomarkers and neuropathy and the differences between type 1 and type 2 diabetics have been scarcely studied [19,21]. In type 1 diabetes, there is an association between adiponectin levels and motor (but not sensory) nerve conduction velocity, whereas in type 2 patients, the adiponectin levels correlate with both motor and sensory nerve velocities after controlling for several confounders (age, sex, time since diagnosis of diabetes, HbA1c, waist circumference, height, total cholesterol, hypertension, current smoking, physical activity, use of lipid-lowering medication, use of nonsteroidal anti-inflammatory drugs, history of myocardial infarction and/or stroke). In type 2 diabetic patients, elevated IL-6 levels in blood associate with the impairment of motor nerve function (but not at the sensory nerve level) and the development of sensorimotor neuropathy in diabetic patients measured at the level of median, ulnar and peroneal motor nerves and the median, ulnar and sural sensory nerves [21]. Adiponectin levels were associated with nerve conduction velocity and diabetic neuropathy in type 1 and 2 diabetes, although in opposite directions, e.g., higher levels of high-molecular weight adiponectin. Total adiponectin and their ratio were associated with prevalent neuropathy and both reduced motor and sensory nerve conduction velocity, whereas no consistent associations were observed for other inflammatory markers such as the concentration in blood of C-reactive protein, IL18, soluble intercellular adhesion molecule-1 and E-selectin. In type 1 diabetes, only high-molecular weight adiponectin and total adiponectin concentrations showed positive associations with motor nerve conduction velocity. Osteoprotegerin (OPG), also known as “osteoclastogenesis inhibition factor”, is a soluble receptor that belongs to the tumor necrosis factor receptor superfamily [50,51]. OPG has a decoy function towards two ligands of tumor necrosis factor receptors: RANKL and TRAIL. Osteoprotegerin acts by binding to RANKL, thereby preventing the interaction of the RANK receptor with its ligand, and inhibiting osteoclast differentiation and survival. It also has an antagonistic role for the apoptosis-inducing ligand receptor TRAIL [23,51]. OPG therefore has an inhibitory effect on osteolysis, and acts as an inhibitor of vascular calcification, which is closely related to osteoporosis and arteriosclerosis. In diabetes, distal symmetric neuropathy is associated with arterial calcification and osteopenia, and these processes are believed to occur due to abnormal functioning of the OPG/RANKL system [52,53]. Plasma osteoprotegerin levels are related to peripheral neuropathy in both types of diabetes, although the strongest relationship is in type 2, and linked to other long-term complications such as peripheral arterial disease and nephropathy [23]. The innate immune response receptor Toll-like receptor 4 (TLR4) belongs to the pattern recognition receptor family, whose activation leads to an intracellular signaling pathway NF-κB and inflammatory cytokine production [54]. There is a strong association of the genetic polymorphisms Asp299Gly/Thr399Ile of TLR4 with reduced prevalence of peripheral neuropathy in type 2 diabetic patients (but not in type 1 diabetic patients) [55] raising the question of whether the innate immune response may be involved in the pathogenesis of neuropathy in type 2 diabetes [56].

### 3.5. Effect of Age and Sex on Neuropathy in Type 1 and 2 Diabetes Patients

The study of the influence of age and gender as independent risk factors in the development of neuropathy in type 1 and type 2 diabetic patients has rarely been addressed by the scientific community. Only six articles [21,23,24,28,32,55] have assessed the contribution of age and gender variables in the development of sensory, motor or autonomous neuropathy by type of diabetes. An advanced age better fits in statistical models for neuropathy severity in type 2 diabetic patients [21,23,24,28,32]. In research conducted by Pan et al. 2019 [28], neither age nor gender were independent factors for the development of cardiac autonomic neuropathy in type 1 diabetic patients. However, an older age was a significant risk factor in the type 2 group. Schamarek et al. 2016 [21], in their study on the association between inflammation biomarkers and sensorimotor neuropathy in diabetics, analyzed the influence of age and gender factors on different variable associations. Among type 1 diabetics, no biomarker was related to the presence of a diabetic sensorimotor neuropathy, but motor nerve conduction velocity (NCV) was inversely associated with C-reactive protein, IL6 and soluble ICAM-1 in an age- and gender-adjusted model, and adiponectin levels were associated with sensory NCV, although the statistical significance of those associations were lost after adjustment for other factors. Among type 2 diabetics, sensorimotor neuropathy was associated with IL6 and adiponectin levels in an age and gender analysis. In a correlation analysis, Arnold et al. 2012 [24] observed that there was no association between different nerve excitability parameters such as determinants of axonal function, and the “age” factor in type 1 diabetics, whereas they found a significant association with type 2 diabetics. The presence of peripheral neuropathy was associated with plasma concentrations of osteoprotegerin in an age- and gender-adjusted model in both type 1 and type 2 diabetics [23]. Pappachan et al. [32] showed that advanced age was a risk factor for the development of cardiac autonomic neuropathy in both types of diabetes, while gender did not suppose an additional risk in any of the groups. Finally, age and gender did not influence the association of diabetic neuropathy and the Asp299Gly and Thr399Ile genotypes in type 2 diabetics, while there was no association in type 1 subjects [55].

## 4. Discussion

Evidence indicates that there are important pathophysiological differences between type 1 and type 2 diabetes, and consequently, it is logical that there may be also differences between the complications derived from each type [4,57]. Due to the differences in the underlying pathology and in the related factors, diabetic polyneuropathy occurs differently in each type of diabetes, causing different functional deficits [57,58,59,60,61]. Studying and knowing these peculiarities is essential for effective prevention, diagnosis, and treatment.

The present study shows that there are differences between type 1 and type 2 diabetes regarding the symptoms of sensory neuropathy. In general, most of the results show a greater impairment of both superficial sensitivity (thermoalgesia) and deep sensitivity (vibration) in type 2 diabetics than in type 1 diabetics. Partanen et al. [62], indicated that the symptoms of sensorimotor neuropathy manifest earlier during the course of type 2 diabetes than they do in type 1 diabetes. Considering this, and looking at the results of this review, it is possible that sensory nervous impairment occurs earlier in type 2 diabetes than in type 1, or that perhaps it may be more acute. However, despite observing a greater impairment of thermal sensitivity in type 2 diabetics, when specifically analyzed, the heat sensitivity was only found impaired in type 1 diabetes [17]. This phenomenon may be explained by the fact that although thermal sensitivity travels through small nerve endings, the fibers responding to heat and cold stimuli are different in terms of their typology and characteristics. Heat sensitivity is transmitted through C-type unmyelinated fibers, while cold is transmitted through Aδ-type myelinated fibers [63,64]. For this reason, each type of thermal nerve fiber may deteriorate differently during the course of the disease. Several papers analyzed in the present study [15,17,19,33] show that the nervous impairment of palestesic sensitivity is greater among type 2 diabetics, or that it predominates in this group, as mentioned above. The reduced sural sensory nerve amplitude in both groups of diabetes is compatible with axonal degeneration, which is more prevalent in the early stages of diabetic peripheral neuropathy [65,66]. Moreover, the nerve conduction rate, which is lower in type 2 diabetics, reflects the impairment of myelin at a later stage [15,21].

Considering the physiology of the somatosensory pathways [63], it is curious that both superficial sensitivity (primarily thermal sensitivity to cold) and deep sensitivity (such as palestesic sensitivity), deteriorate mainly in type 2 diabetes, since they travel through different sensory pathways. However, if we look at the nervous anatomy of these pathways, we can see that the short fibers that transmit cold are myelinated, while those that transmit vibration are large fibers that are also myelinated [67]. The fact that the sensitivity that travels through myelin fibers is more affected in type 2 diabetics is an interesting issue that should be resolved in future research.

Muscle weakness is one of the later complications of diabetic neuropathy, and according to the evidence, there is a relationship between reduced muscle strength and peripheral motor nerve dysfunction [68,69,70]. In our review, only one study has compared motor nerve impairment between type 1 and type 2 diabetes patients. It examined the association between the deterioration of muscle strength and the type of diabetes, and concluded that there were no differences between the two types of diabetes [15]. The lack of studies with which to compare this result prevents a complete analysis for establishing a conclusion in this regard. It has been suggested that continuous exposure to high concentrations of glucose causes early metabolic alterations in the nerves and a decline in Na+/K+-ATPase activity, as well as an impairment of microvascular perfusion [71,72,73]. Likewise, it appears that the axonal function was altered in patients with type 1 diabetes, but the patients did not present symptoms of motor neuropathy [24]. It is possible that an early approach may prevent the development of motor neuropathy with irreversible damage [24,62]. 

Cardiac autonomic neuropathy (CAN) is one of the diabetic complications with the greatest impact at a clinical level, but is nevertheless one of the most underdiagnosed [61,74]. According to the published studies, the prevalence of CAN appears to be variable in both types of diabetes. While it ranges between 2 and 91% in type 1 diabetes, it ranges between 25 and 75% in type 2 diabetes [40,75]. The fact that several different tests are used to diagnose this condition and the lack of consensus in its diagnosis may be the cause of undiagnosed cases and consequently, considerable variability in its prevalence [74]. According to the study by Pan et al. 2019 [28], the most sensory way to diagnose CAN is by a combination of certain tests; for type 1 diabetics it is the Valsalva maneuver together with the lying-to-standing test, for type 2 diabetics it is the combination of deep-breathing and the Valsalva test. In our findings, we observed that there is a greater sympathetic than parasympathetic impairment, as indicated by the screening tests for autonomic cardiac neuropathy [15,17,19,28,29,31,32,33,35]. These results are consistent with the research of Pourmoghaddas and Hekmatnia [76] who confirmed that parasympathetic abnormality was three times greater than the sympathetic affectation in diabetic CAN. Furthermore, tests that examine parasympathetic activity, such as HR response 15 and 30 s after changing position, HR response during deep respiration and HR response during Valsalva maneuver, were found to be more impaired in type 2 diabetics [15,17,19,33], with the exception of the HR variation test during rest, which was more affected in type 1 diabetics [17,19]. These results seem to indicate that the most altered autonomic nervous component in CAN is the parasympathetic system, and that it is altered above all in type 2 diabetics. The association between autonomic cardiac neuropathy and retinopathy in diabetic subjects [35] has been verified in other studies in type 1 diabetics [77] and in type 2 diabetics [78]. The correlation between CAN and hypertension has been well documented. These findings suggest that the possible cause of this association is sympathetic hyperactivity and a loss of parasympathetic regulation, which lead to an increase in the heart rate [79,80]. The high prevalence of CAN in type 2 diabetes, can synergize with the adverse effects on cardiovascular system induced by other common comorbidities such as hypertension, dyslipidemia and obesity [81,82,83] which contribute to premature morbidity and mortality [84,85,86]. In our review, the prevalence of erectile dysfunction was higher in type 2 diabetics. As for other disorders derived from autonomic neuropathy, erectile dysfunction is a typical complication of diabetes [87,88,89,90], but only two studies compared it in both types of diabetes [26,27]. Further studies are also needed to compare disorders such as gastroparesis and gallbladder function of neuropathic origin in the two types of diabetes [33,34].

One of the modifiable factors that may be related to the development of diabetic neuropathy, especially in people with diabetes, is obesity, which is an entity that is often comorbid, especially in type 2 diabetic patients [19,31]. It is well-know that autonomic nervous system dysfunction has a bidirectional relationship with obesity [91]. On the one hand, there is good scientific evidence that alterations of the autonomic nervous system could be involved in the pathogenesis of obesity, and on the other hand, obesity (especially central, visceral obesity) induces autonomic nervous system dysfunction, which may be involved in hemodynamic and metabolic alterations that increase the cardiovascular risk of obese individuals such as the insulin resistance, among others, which increases the risk of type 2 diabetes [91,92]. According to this, the autonomic neuropathy in diabetics was more prevalent in obese patients. Regarding the incidence of cardiac autonomic neuropathy, it was higher in obese patients with type 2 diabetes which also adds higher odds to present comorbid arterial hypertension [29,31]. The incidence of erectile dysfunction and alterations in fasting gallbladder volume and less complete emptying increased with increasing body-mass index [27,34]. In contrast to autonomic neuropathy, BMI seems to not play a significant role on motor nerves alterations in diabetic patients [24], however much work should be done regarding sensorimotor neuropathy and obesity, taking into account that weight reduction significantly improved autonomous dysfunction in obesity and likely autonomic neuropathy in diabetes [91]. 

Regarding peripheral biomarkers associated to neuropathy, some of those are related to chronic inflammatory state such as the inflammatory cytokine IL-6 [93], and to the innate immune response receptor TLR4 [55] associated more frequently to the severity of neuropathy in type 2 diabetes patients. In type 1 diabetes, the most promising biomarker related to neuropathy appearance and severity is adiponectin, the richest adipokine in human plasma mainly secreted from white adipose tissue. Changes in the concentration of adiponectin isoforms in blood have also demonstrated in type 2 diabetes [94]. These differences in peripheral biomarkers might contribute to the pathophysiological progression of various diabetic neuropathy types and symptoms and it clearly deserves further studies.

The increase in life expectancy worldwide in recent decades, particularly among women, has meant that people with diabetes live longer with the disease and therefore suffer from its complications, with the most prevalent being diabetic neuropathy [95,96]. As a result, it is important to study age and gender as risk factors involved in the development of diabetic neuropathy. However, only six articles in this study have examined these issues. In their study, Grisold et al. [97] recently pinpointed that age and gender are possible risk factors for the development of diabetic peripheral neuropathy in type 1 and type 2 diabetes, although their exact contribution is unknown. After reviewing the articles, age was observed to be a risk factor in the development of autonomic cardiac neuropathy and motor neuropathy, with the latter measured in parameters of motor nervous excitability, and in both cases this relationship was only found in type 2 diabetics [24,28]. However, a relationship was detected between CAN and the “advanced age” factor in both type 1 and type 2 diabetic patients [32]. These results suggest that older age, which is a more typical characteristic in studies of type 2 diabetics, is a risk factor for diabetic neuropathy. With regard to the influence that gender could have in this aspect, little research has been done and no relationship has been found.

Diabetic neuropathy is the most common complication of diabetes mellitus and the one that has the greatest impact on the life of the patient, with high rates of morbidity and mortality. Knowing how sensory, motor, and autonomic neuropathy affects each type of diabetes and their differences and similarities is essential for developing an adapted diagnostic and therapeutic plan in order to better address the disease and avoid irreversible damage.

This review has identified several aspects that require future research. One of the unknown factors identified is the different involvement of the nerve fibers responsible for each sensory function, and its possible relationship with its characteristic anatomy and physiology function or dysfunction in diabetes. Motor neuropathy and its peculiarities, typical of type 1 and type 2 diabetes, require a great deal of attention since it is the least investigated neuropathy domain investigated so far. As for autonomic neuropathy, it is important to clarify the mechanism of sympathetic and parasympathetic involvement in both types of diabetes, as well as the related disorders to alterations or autonomic nervous system. In this way, the characterization of the disease subtype will improve the diagnosis and treatment of neuropathy and it will consequently allow a more personalized and effective treatment besides glycemic index control.

## Figures and Tables

**Table 1:** Differences between type 1 (T1) and type 2 (T2) diabetes in sensory neuropathy.

Reference	Population Characteristics			Measurement of Neuropathy	Main Outcomes
Balducci et al. 2014 [15]	N	400		Questionnaire Michigan Neuropathy Screening Instrument [16] Diapason and biothesiometer Monofilament	The vibration perception threshold in the malleolus and hallux, was higher in type 1 diabetics than in type 2 diabetics. No differences were found in amplitude, conduction velocity and distal latency of sural sensory nerve, between type 1 and type 2 diabetics.
	Mean duration of DM	T1	22.1 ± 11.1		
		T2	14.0 ± 9.0		
	Level of glycemic control (HbA1c) (%)	T1	7.44 ± 1.38		
		T2	7.08 ± 1.45		
	Gender (%)	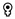	43%		
		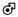	57%		
	Age (mean)	61.9 ± 11.1			
Meyer et al. 2003 [17]	N	42		Pain and thermal sensitivity tester [18] Diapason	The frequency of cold sensitivity impairment was higher in type 2 diabetics than in type 1 (60% vs. 40%) The impairment of heat sensitivity was only found in 10% of type 1 diabetics. The frequency of vibration sensitivity impairment was higher in type 2 diabetics than in type 1 (45% vs. 15%) The impairment of pain sensitivity was not found in neither group of diabetics.
	Mean duration of DM	T1	7.8 ± 2.3		
		T2	10.4 ± 2.4		
	Level of glycemic control (HbA1c) (%)	T1	9.4 ± 0.6		
		T2	8.3 ± 0.3		
	Gender (%)	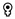	54.8%		
		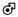	45.2%		
	Age (mean)	46.3 ± 2			
Aulich et al. 2019 [19]	N	198		Neurosensory Analyzer model TSA-II [20] →Thermal sensitivity for cold and hot at the left foot dorsum. →Vibratory sensitivity at the left malleolus and left great toe.	The prevalence of sensory neuropathy was higher in type 2 diabetics than in type 1 diabetics.
	Mean duration of DM	T1	8.1		
		T2	1.8		
	Level of glycemic control (HbA1c) (%)	T1	8.5		
		T2	6.6		
	Gender (%)	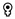	49%		
		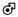	51%		
	Age (mean)	15.5 ± 2.4			
Schamarek et al. 2016 [21]	N	513		Questionnaire Neuropathy Disability Score and Questionnaire Neuropathy Symptom Score [22] Electromyography	The prevalence of sensory neuropathy was higher in type 2 diabetics which was reflected in a lower sensory nerve conduction rate. Neuropathy Disability Score (NDS) and Neuropathy Symptom Score (NSS) were also more altered in type 2 diabetics.
	Mean duration of DM	T1	Not reported		
		T2	Not reported		
	Level of glycemic control (HbA1c) (%)	T1	6.91 ± 1.70		
		T2	6.53 ± 1.09		
	Gender (%)	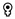	35.5%		
		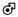	64.5%		
	Age (mean)	47.4 ± 11.2			
Nybo et al. 2009 [23]	N	505		Semmes–Weinstein monofilament	Neuropathy was more prevalent in type 1 diabetic patients.
	Mean duration of DM	T1	42–43 approx.		
		T2	4.2–5.7 approx.		
	Level of glycemic control (HbA1c) (%)	T1	7.5–7.8 approx.		
		T2	7.3–7.5 approx.		
	Gender (%)	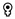	43%		
		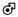	57%		
	Age (mean)	58.3 ± 10.4			

PN: peripheral neuropathy.

**Table 2:** Differences between type 1 (T1) and type 2 (T2) diabetes in motor neuropathy.

Reference	Population Characteristics			Measurement of Neuropathy	Main Outcomes
Balducci et al. 2014 [15]	N	400		Questionnaire Michigan Neuropathy Screening Instrument [16] Dynamometer: muscle contraction of shoulders and lower limbs. Electromyography: →Conduction velocity, amplitude, and distal latency of motor peroneal nerve and sensory sural nerve.	Muscle strength was higher in type 1 diabetics than in type 2 diabetics.
	Mean duration of DM	T1	80		
		T2	14.0 ± 9.0		
	Level of glycemic control (HbA1c) (%)	T1	7.44 ± 1.38		
		T2	7.08 ± 1.45		
	Gender (%)	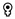	43%		
		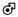	57%		
	Age (mean)	61.9 ± 11.1			
Schamarek et al. 2016 [21]	N	513		Questionnaire Neuropathy Disability Score and Questionnaire Neuropathy Symptom Score [22] Electromyography: →Conduction velocity of medium, ulnar, and peroneal motor nerves.	The prevalence of motor neuropathy was higher in type 2 diabetics. Neuropathy Disability Score (NDS) and Neuropathy Symptom Score (NSS) were also more altered in type 2 diabetics.
	Mean duration of DM	T1	161		
		T2	Not reported		
	Level of glycemic control (HbA1c) (%)	T1	6.91 ± 1.70		
		T2	6.53 ± 1.09		
	Gender (%)	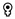	35.5%		
		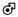	64.5%		
	Age (mean)	47.4 ± 11.2			
Arnold et al. 2013 [24]	N		40	Total Neuropathy Score [25] Electromyography: →Conduction velocity of motor nerves Nerve excitability testing: →Compound muscle action potentials of median motor nerve at the wrist	Abnormalities in nerve excitability parameters were found in type 1 diabetes patients.
	Mean duration of DM	T1	7.78 ± 1.33		
		T2	8.07 ± 1.14		
	Level of glycemic control (HbA1c) (%)	T1	7.64 ± 0.33		
		T2	7.70 ± 0.39		
	Gender (%)	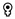	27.5%		
		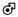	72.5%		
	Age (mean)	41			

**Table 3:** Differences between type 1 (T1) and type 2 (T2) diabetes in autonomic neuropathy.

Reference	Population Study Characteristics			Measurement of Neuropathy	Main Outcomes
Balducci et al. 2014 [15]	N	400		CV autonomic reflex tests: heart rate variation during rest, to deep breathing, to cough test, to standing, systolic blood pressure falls on standing.	No differences were found for the systolic blood pressure response to standing, between type 1 diabetics and type 2 diabetics. Heart rate response to deep breathing was higher in type 1 diabetics than in type 2. Heart rate response to standing was higher in type 1 diabetics than in type 2. No differences were found between both groups of diabetics in heart rate variation during rest, and in cough test.
	Mean duration of DM	T1	22.1 ± 11.1		
		T2	14.0 ± 9.0		
	Level of glycemic control (HbA1c) (%)	T1	7.44 ± 1.38		
		T2	7.08 ± 1.45		
	Gender (%)	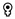	43%		
		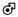	57%		
	Age (mean)	61.9 ± 11.1			
Meyer et al. 2003 [17]	N	42		CV autonomic reflex tests: heart rate variation during rest, to deep breathing, to Valsalva maneuver. Peripheral sympathetic test: laser doppler anemometry for vasomotion assessment.	In type 1 diabetes patients, vasomotion impairment was more common in subjects with autonomous neuropathy than in those without it. In contrast, in type 2 diabetics group, no differences were found in deterioration of vasomotion between subjects with and subjects without autonomic neuropathy. Type 1 diabetics who did not suffer autonomic neuropathy had vasomotion amplitudes greater than those with autonomic neuropathy while in type 2 diabetics, no differences were observed. In both type 1 and type 2 diabetics, a positive association was found between vasomotion amplitudes and Valsalva ratio. There no was correlation between vasomotion amplitudes and variation heart rate during deep breathing test, in neither group.
	Mean duration of DM	T1	7.8 ± 2.3		
		T2	10.4 ± 2.4		
	Level of glycemic control (HbA1c) (%)	T1	9.4 ± 0.6		
		T2	8.3 ± 0.3		
	Gender (%)	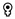	54.8%		
		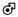	45.2%		
	Age (mean)	46.3 ± 2			
Fedele et al. 1998 [26]	N	9868		Interview about presence or absence of erectile dysfunction (achieving and maintaining a sufficient erection for a satisfactory sexual relationship). Review of the medical history	The prevalence of erectile dysfunction was much higher in type 2 diabetics than in type 1 diabetics.
	Mean duration of DM	T1	Not reported		
		T2	Not reported		
	Level of glycemic control (HbA1c) (%)	T1	Not reported		
		T2	Not reported		
	Gender (%)	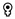	0%		
		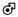	100%		
	Age (range)	20–69			
Fedele et al. 2001 [27]	N	1010		Interview about presence or absence of erectile dysfunction (achieving and maintaining a sufficient erection for a satisfactory sexual relationship).	The incidence of erectile dysfunction over a follow-up period of 2.8 years was 1.6 times higher in type 2 diabetics than in type 1 diabetics.
	Mean duration of DM	T1	Not reported		
		T2	Not reported		
	Level of glycemic control (HbA1c) (%)	T1	Not reported		
		T2	Not reported		
	Gender (%)	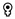	0%		
		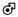	100%		
	Age	19–79			
Aulich et al. 2019 [19]	N	198		Variability of basal heart rate during 10 minutes of supine decubitus by ECG.	The prevalence of cardiac autonomous neuropathy was higher in type 2 than in type 1 young diabetics.
	Mean duration of DM	T1	8.1		
		T2	1.8		
	Level of glycemic control (HbA1c) (%)	T1	8.5		
		T2	6.6		
	Gender (%)	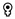	49%		
		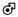	51%		
	Age (mean)	15.5 ± 2.4			
Pan et al. 2019 [28]	N	2.048		CV autonomic reflex tests: heart rate response to deep breathing, heart rate response to Valsalva maneuver, to standing systolic blood pressure response to standing	The prevalence of cardiac autonomous neuropathy was similar between type 1 and type 2 diabetics. In type 1 diabetics, the optimal diagnostic strategy for CAN was the combination of the variation in HR during the Valsalva maneuver and in response to standing. In contrast, in type 2 diabetics, the HR variation test in response to deep breathing had great sensitivity and the best combination was with the Valsalva maneuver.
	Mean duration of DM	T1	Not reported		
		T2	Not reported		
	Level of glycemic control (HbA1c) (%)	T1	Not reported		
		T2	Not reported		
	Gender (%)	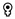	50%		
		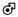	50%		
	Age (mean)	58.86 ± 10.8			
Gulichsen et al. 2012 [29]	N	323		CV autonomic reflex tests using a new handheld device Vagus^TM^ [30]: heart rate variation during rest, heart rate response to deep breathing, to Valsalva maneuver, to standing.	The prevalence of cardiac autonomous neuropathy was higher in type 2 diabetics than in type 1 diabetics.
	Mean duration of DM	T1	Not reported		
		T2	Not reported		
	Level of glycemic control (HbA1c) (%)	T1	Not reported		
		T2	Not reported		
	Gender (%)	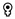	46.1%		
		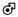	53.9%		
	Age (mean)	56.1 ± 11.4			
Ayad et al. 2010 [31]	N	310		Questionnaire of symptoms CV autonomic reflex tests: heart rate response to deep breathing, to standing, and to Valsalva maneuver. Systolic blood pressure response to standing.	No differences were found between the diabetic groups regarding the prevalence of postural hypotension. The prevalence of neuropathy did not show significant differences between type 1 diabetics and type 2 diabetics, although in this latter group, it was slightly higher. Among type 2 diabetics, neuropathy was more severe in those treated with insulin compared to those treated with oral antidiabetics.
	Mean duration of DM	T1	9.8 ± 7.7		
		T2	7.4 ± 13.6		
	Level of glycemic control (HbA1c) (%)	T1	10.4 ± 2.7		
		T2	9.8 2.5		
	Gender (%)	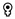	53.2%		
		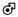	46.8%		
	Age (mean)	41.7 ± 12.8			
Pappachan et al. 2008 [32]	N	100		CV autonomic reflex tests: Heart rate during rest, to deep breathing, to Valsalva maneuver. Systolic blood pressure response to standing. Assessment of diastolic blood pressure response during sustained handgrip. Determination of QTc interval (ECG)	The maximum systolic and diastolic BP during exercise were not different between patients with retinopathy and patients without retinopathy, in neither diabetic group. Recovery of systolic and diastolic BP at 2 minutes post exercise was not different between those diabetics with retinopathy and those without, in the group of type 1 diabetics. In contrast, in type 2 diabetics, the recovery of SBP was higher in subjects with retinopathy, while this difference was not observed for DBP. In both groups of diabetics, univariate analysis showed a significant and positive correlation between severity of CAN and prolongation of QTc interval.
	Mean duration of DM	T1	Not reported		
		T2	Not reported		
	Level of glycemic control (HbA1c) (%)	T1	Not reported		
		T2	Not reported		
	Gender (%)	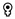	60%		
		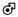	40%		
	Age (Median)	53			
Koçkar et al. 2002 [33]	N	40		Determination of QTc interval (ECG)	In both diabetic groups, QTc dispersion was longer than in healthy controls, but this difference only was significant in type 2 diabetics. Gastric emptying time was significantly higher in type 1 and type 2 diabetics than healthy controls.
	Mean duration of DM	T1	Not reported		
		T2	Not reported		
	Level of glycemic control (HbA1c) (%)	T1	Not reported		
		T2	Not reported		
	Gender (%)	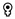	35%		
		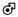	65%		
	Age	39.5 ± 9.3			
Palasciano et al. 1992 [34]	N	21		Electromyography: Function of the striatum muscle. Conduction velocity and potential amplitude of deep peroneal motor nerves. Ultrasound for evaluate gallbladder volume and study motor function (motility), during fasting and after intake.	Motor function of gallbladder was not correlated with type of diabetes.
	Mean duration of DM	T1	Not reported		
		T2	Not reported		
	Level of glycemic control (HbA1c) (%)	T1	Not reported		
		T2	Not reported		
	Gender (%)	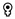	52%		
		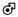	48%		
	Age (mean)	50 ± 15			
Kramer et al. 2008 [35]	N	112		Exercise electrocardiography (at different intervals): heart rate and blood pressure at rest and estimated workload in metabolic equivalents (METs). CV autonomic reflex tests: heart rate response to Valsalva maneuver, beat-to-beat heart rate variation. Heart rate response to standing. Postural fall in blood pressure. Assessment of diastolic blood pressure response during sustained handgrip.	Autonomous cardiac neuropathy, detected by tests of variability of HR with exercise, was related to the development of retinopathy in subjects with type I and type II diabetes.
	Mean duration of DM	T1	21.5–27 approx.		
		T2	8.7–10.7 approx.		
	Level of glycemic control (HbA1c) (%)	T1	Not reported		
		T2	Not reported		
	Gender (%)	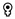	43%		
		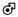	57%		
	Age (mean)	51.6 ± 9			

CV: cardiovascular; ECG: electrocardiogram; CAN: cardiac autonomic neuropathy; HR: heart rate; BP: blood pressure; SBP: systolic blood pressure; DBP: diastolic blood pressure; QTc: the QT interval corrected for heart rate.

## Data Availability

Not applicable.
